# The Promiscuity of Disulfiram in Medicinal Research

**DOI:** 10.1021/acsmedchemlett.3c00450

**Published:** 2023-11-07

**Authors:** Boris Cvek

**Affiliations:** Palacky University, Univerzitni 22, Olomouc 771 11, Czech Republic

**Keywords:** disulfiram, panacea, in vitro, promiscuity, repurposing

## Abstract

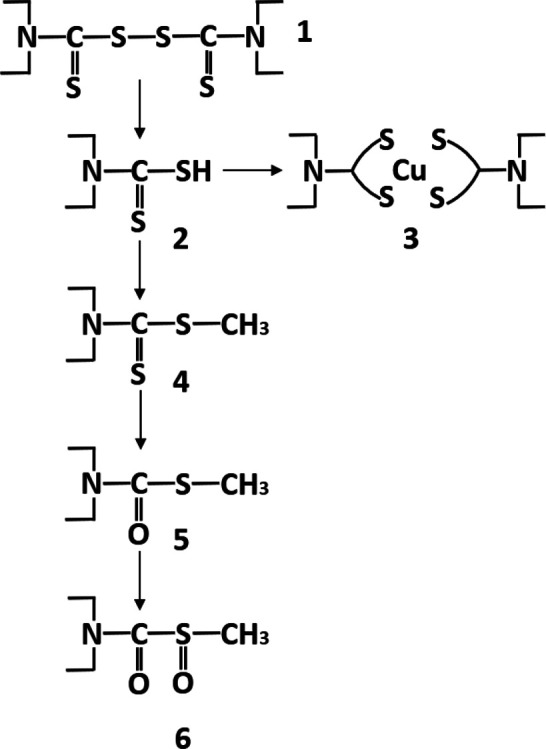

Recent efforts to repurpose disulfiram, a drug used in alcohol-aversion therapy for decades, for other diseases suggest the molecule is almost an *in vitro* panacea: it seems to be effective against various cancers (by multiple mechanisms of action), Alzheimer’s disease, obesity and metabolic syndrome, pythiosis, lyme borreliosis, COVID-19, and sepsis. The problem is that the molecule almost does not exist in the body after ingestion and, most importantly, is not the pharmacologically active entity in alcoholic patients, being rather a prodrug. This prodrug is widely and misleadingly used in many *in vitro* and *in vivo* experiments regardless of its physiologically reachable concentration or its metabolism *in vivo*.

There are hundreds of studies in the scientific literature using disulfiram (**1** in Figure 1) *in vitro* or in animals to demonstrate its ability, in a medical sense, to alter a protein of interest in various cells. Indeed, for the period 2010–2022, there are more than 200 published articles—reviews, abstracts, letters, etc. are excluded—on “disulfiram AND cancer AND *in vitro*” in the Web of Science database. Although those efforts are widely motivated by repurposing of the already approved drug, i.e., **1**, as an active compound in the treatment of alcoholism, they often ignore the basic facts about the metabolism and pharmacodynamics of the molecule in the human body or the effects of the several known metabolites. The molecule is highly reactive, and its effects *in vitro* may, in fact, be due to the activity of unidentified products of its chemical reactions in culture media or in cells. This is reflected by the remarkable variety of mutually contradictory or non-related mechanisms of action of **1** in cancer and also, maybe more tellingly, by a growing list of diseases that **1** is suggested to treat—like a new incarnation of a panacea.

**Figure 1:**
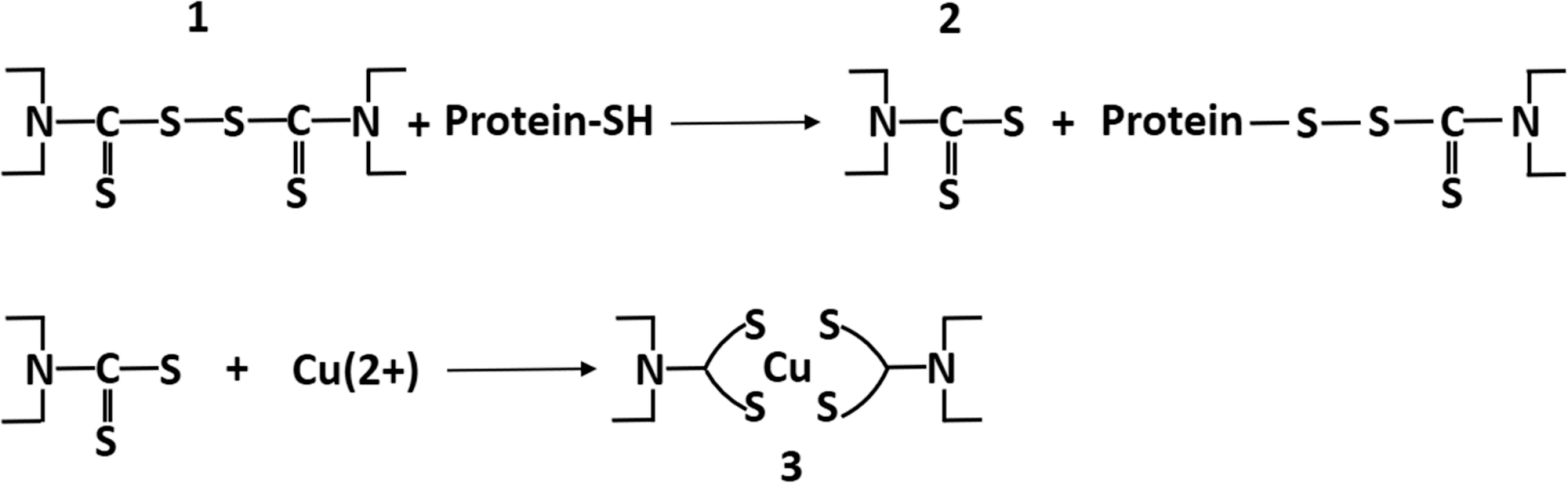
Basic chemistry of **1**.

## The Drug in the Clinic

Dithiocarbamates and thiuram (di)sulfides are well known and highly reactive compounds that have had applications in analytical chemistry, agriculture, and biomedical investigations for many decades.^1^ Disulfiram (**1**) was used at first to accelerate vulcanization of rubber, then as a medication for scabies, and eventually was shown, by serendipity, to deter alcoholic patients from further drinking. A pair of Danish researchers in the 1940s, Jacobsen and Hald, investigated the drug as a treatment against intestinal parasites on the basis of the evidence that it chelated copper, and after obtaining promising results in rabbits, they decided to test the drug by ingesting it. To their surprise, they realized they had unpleasant experiences after drinking alcohol, which they considered as a serious side effect, preventing further development of the drug. Paradoxically, in later years and by chance, this negative side effect became the reason for use of the drug in the treatment of alcoholism.^2^ Under the trade name Antabuse, **1** has been used in therapy for alcoholism for more than 70 years, with a currently recommended dosing of 250–500 mg/day for several months.^3^

**Figure 2:**
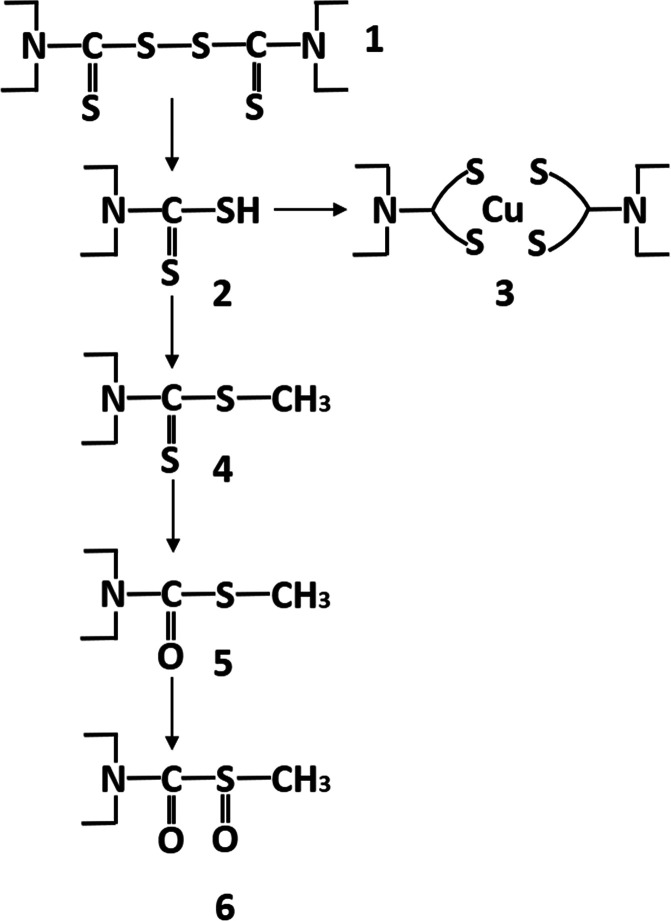
Metabolism of **1**.

After ingestion of **1**, aldehyde dehydrogenase (ALDH) is inhibited, but to inhibit ALDH *in vivo*, **1** must be metabolized to **6** (see below and Figure 2). When ALDH is inactivated, metabolism of ethanol cannot proceed from acetaldehyde to acetic acid, and the accumulation of acetaldehyde in the body after the consumption of alcoholic beverages is responsible for the alcohol-aversion symptoms of the therapy.^4^ An person with alcoholism using **1** can, after drinking ethanol-containing beverages, experience various unpleasant symptoms, from sweating and headache to nausea, vomiting, or even life-threating events. Drinking ethanol-containing products is thus effectively transformed into rather a deterring experience, and this causes the person using **1** to have an aversion to drinking. In the absence of ethanol, side effects of the drug are usually mild, the most serious being hepatotoxicity, which is mostly reversible.^5^ Antabuse is an inexpensive medication available in many countries around the world.

## Repurposing the Drug in Clinical Trials

Repurposing **1** or its metabolite **2** (Figure 2) for cancer and AIDS has been a continuous endeavor in biomedical research, spanning back to the 1970s and 1980s, respectively.^6^ It is worth noting two randomized, phase II, placebo-controlled and blinded clinical trials conducted in cancer patients. In 1993, the results of a study of **2** as an adjuvant therapy in high-risk breast cancer patients were published: at 5 years, overall survival was 81% in patients taking **2**, using a low 10 mg/kg dosage of the drug administered *per os* once a weak, compared to 55% in the placebo group.^7^ In spite of many research publications on the mechanism of action of **1** toward cancer cell lines *in vitro*, 30 years after the results were published, we still do not know (with the exception of a hypothesis that the active compound is a copper complex known as CuET (**3**); see below and Figure 1) the mechanistic underpinning of the effect of **2** in breast cancer, except for the simple fact that **1** cannot be the active compound *in vivo*. More recently, another clinical study tested the addition of a low dose of **1** to chemotherapy, in this case in patients with metastatic lung cancer. An increase of survival was noted (10 vs 7.1 months in active vs placebo group), and two long-term survivors were both in the group of patients administered with **1**.^8^

## Basic Chemistry of the Molecule

**1** easily forms mixed sulfides with SH-containing compounds, which may lead to inhibition of various enzymes in experiments with purified biomolecules or even in the cells (Figure 1). According to a review from 1981, “it is possible that disulfiram inhibits more or less all −SH enzymes and cofactors with −SH groups.” ^4^ A telling example is ALDH itself: **1** inhibits purified human ALDH^9^ but—as shown below—has to be metabolized to achieve the same effect *in vivo*. Although dithiocarbamates display rich coordination chemistry with transition metals in the laboratory,^10^ only the copper complex **3** has been demonstrated to be a stable metabolite of **1** in mice and humans so far (Figure 1).^11^ Furthermore, it also has been demonstrated that, in copper-containing media used to culture cancer cells, **1** reacts with copper to form the complex **3**, which is the active compound that kills the cells (not **1** itself).^12^

## The Physiologically Achievable Concentration

**1** is rapidly (in 4 min) reduced to its metabolite **2** in blood, where **2** itself is not stable for a long time, with a half-life of about 100 min.^13^ The pharmacokinetic profile of **1** in alcoholic patients following single or repeated oral doses of 250 mg/day was determined in 1980s. The peak plasma concentration of **1** was reached after 9 h at about 0.4 μg/mL, i.e., under 1.5 μmol/L (the molar mass of **1** is 296.54 g/mol).^14^ More recently, the pharmacokinetics of **1** have been studied in HIV-1-infected patients receiving 500 mg (the highest recommended dosing), 1000 mg, or 2000 mg of the drug for three consecutive days (10 patients per each dosing scheme). The plasma concentrations in almost all of the patients taking 500 mg of **1** per day did not reach 100 ng/mL (i.e., 0.1 μg/mL, or about 0.34 μM; see Table 1). The highest plasma concentration was observed in four or five patients on the highest dose and amounted to between 0.5 and 0.6 μg/mL (under 2 μM).^15^ These numbers are a result of the state-of-the-art measurement in the last years of the second decade of the 21st century, but a similar finding is known from a report published in 1988, which determined the concentration of **1** in patients on therapeutic doses of the drug: “Detectable concentrations in the range of 0.1–0.2 micromoles per liter could not be obtained until the second week of treatment.” ^16^ Importantly, these data are in sharp contrast with concentrations used widely *in vitro*, which are often above 2 μM when repurposing the drug for new indications, as discussed below. I must conclude that any concentration of **1** above 0.34 μM used in biomedical experiments is too high to be achievable in real patients at recommended dosing.

**Table 1:** Highest Peak Plasma Concentrations of **1** and Its Metabolites **2**, **4**, and **6** in Patients

	peak plasma concn (approx.) for **1**, **2**, **4**, and **6**	
dose of **1** (per day for 3 days)	ng/mL	μmol/L
500 mg	100, 120, 90, 70	0.34, 0.81, 0.55, 0.43
1000 mg	300, 300, 100, 110	1, 2, 0.61, 0.67
2000 mg	600, 600, 150, 200	2, 4, 0.92, 1.23

## The Active Compound in Treating Alcoholism

**1** itself is not the pharmacologically active compound in patients undergoing the alcohol-aversion therapy described above. Pretreatment of rats with a cytochrome P450 inhibitor prevented ALDH inhibition by **1** and its metabolites **2**, **4**, and **5** (Figure 2) suggesting that even metabolites of **1** must be metabolized to achieve the therapeutic effect of the drug.^17^ All of the metabolites were potent ALDH inhibitors in rats, but only the further oxidized metabolite **6** (Figure 2), did not require activation by cytochrome P450s. Moreover, **6** was a potent inhibitor of rat liver ALDH both *in vitro* and *in vivo*.^18^ When another compound, diethyldithiocarbamate methyl ester sulfine (DMES), was administered to rats, inhibition of ALDH and an alcohol-aversion reaction was observed, although this compound was not able to inhibit the enzyme *in vitro*. The major metabolite of DMES in rats was **5**(19) which is further metabolized to the sulfoxide, which may be metabolized to the sulfone, both potent ALDH inhibitors.^20,21^ To sum up, the exact mechanism of ALDH inhibition by the metabolites of **1***in vivo* is still not known, but most probably, as determination of adducts of mitochondrial ALDH from mice treated with disulfiram demonstrated, the inhibition of ALDH “occurred by carbamoylation caused by one of the disulfiram metabolites”, most likely by **6**.^22^

## “Targeting” ALDH in Cancer

**Figure 3:**
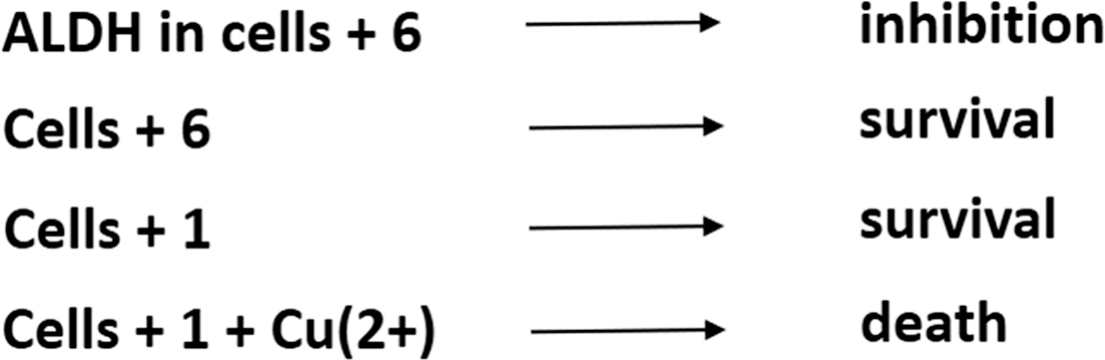
Inhibition of ALDH in cancer cells is not the cause of toxicity of **1**.

Not surprisingly, many proteins and pathways have been suggested to be targeted by **1** in cancer cells *in vitro*. Typically, publications reporting this have neither a mention of the general situation in the field nor an attempt to reconcile their own results with other proposed mechanisms of action. They regularly use micromolar or higher concentrations of the drug. To mention an illustrative example of repurposing of **1** for cancer, the influential theory that inhibition of ALDH by **1** is the cause of its anti-cancer effect should be briefly discussed (Figure 3). If the hypothesis were to be tested seriously, **6** would have to be included in such studies—but, surprisingly, only one publication has appreciated that aspect of the biochemical pharmacology of that compound.^12^ The authors demonstrated that **6** is, indeed, a potent inhibitor of the enzyme, but it is not able to kill cancer cells. On the other hand, the ability of **1** to suppress the cells was caused by **3**, which is the product of the aforementioned chemical reaction of **1** with copper ions in the cell culture medium; if there is no copper ion in the medium, **1** is not able to kill the cells. When ALDH activity is measured in dead cells—after a long period of treatment with **1** in copper-ion-containing media—the enzyme seems to be “inhibited” by the compound. As another study has shown,^11^**3** is also present in the blood of people with alcoholism taking **1** and in tumor xenografts of mice fed with the drug. The unique mechanism of action of **3** to destroy cancer cells—inhibition of p97-mediated degradation of ubiquitinated proteins—is, importantly, the same *in vitro* as it is *in vivo* at physiologically relevant concentrations.

## A Panacea *In Vitro*

It is clear that meaningful *in vitro* experiments building on the fact that **1** is already approved and has been, for a long time, used as a drug for the treatment of alcoholism must take into account its metabolism in the body, i.e., use its real, defined metabolites in the test tube (e.g., **3** or **6**). If this is ignored, as usually is the case, **1** may be masquerading as a panacea under *in vitro* conditions. Indeed, judging from the recent scientific literature, **1** seems to be a promising treatment for conditions as distinct as Alzheimer’s disease, obesity and metabolic syndrome, pythiosis, Lyme borreliosis, and even the current coronavirus pandemic. For example, two recent studies in *Nature* and *Nature Immunology* suggest that **1** could treat COVID-19 and sepsis, respectively (see references below).

### Alzheimer’s Disease^23^

In this publication, the authors screened an FDA-approved drug library and identified **1** as a novel ADAM10 gene expression enhancer. In fact, **1** increased ADAM10 production in human neuronal cells *in vitro* and in peripheral blood cells in a mouse model of Alzheimer’s disease. **1** also reduced plaque burden in the dentate gyrus and ameliorated behavioral deficits of the mice. The authors did not demonstrate the presence of **1** in the mice and did not discuss its metabolism regarding physiological significance of the concentrations they used (up to 5 μM). The original reason to use **1** was clearly its presence in an FDA-approved drug library.

### Obesity and Metabolic Syndrome^24^

This work presented a follow-up of an *in vivo* study showing a potent protective effect of **1** in mice against obesity. When the authors explained the effect by *in vitro* models, they used concentrations of **1** at 20 μM or even higher. They did not discuss the metabolism of the drug or the relevance of the concentrations they selected and used.

### Pythiosis^25^

Here, a total of 27 *Pythium insidiosum* strains were found to be susceptible to **1** in a dose-dependent manner: the minimal concentration to be effective was 8 mg/L (compare with the peak serum concentration of **1** at the highest recommended dose, which is 0.1 μg/mL = 0.1 mg/L; see Table 1). Moreover, the authors of this study presented models of molecular docking of **1** and interactions of the molecule and the predicted binding sites in urease and ALDH, respectively. The authors discussed “attempts” to measure the serum concentration of **1** from the data published in the 1970s and speculated that the peak serum concentration may be higher than 8 mg/L. They did not mention the detailed measurements of the pharmacokinetics of **1** made available in the later scientific literature. Although the authors dealt with the metabolism of **1** and demonstrated by molecular docking that some metabolites of **1**, **2** and diethylamine, are capable of binding urease and ALDH, they did not mention **6**. Instead, the authors stated that **1** “inhibits the growth of many pathogenic microorganisms, including *P. insidiosum*, by inactivating several proteins.”

### Lyme Borreliosis^26^

A study conducted in mice suggested that **1** is a potent drug against the disease. But the *in vivo* effect should rather be explained on the basis of the activity of its metabolites, because *in vitro* the active concentration of the drug in this publication was well above 0.5 μM. The observed behavior of the drug was summed up as follows: “At low concentrations (ranging from 0.625–10 μM), the disulfiram in DMSO and disulfiram in cyclodextrin drugs concentration–response profile is sigmoidal. In contrast, at higher concentrations (ranging from 25–100 μM), the disulfiram drugs lost their efficacy and exhibited a U- or bell-shaped curve.” This loss of the efficacy of **1** was attributed by the authors “to inadvertent effects arising from the colloidal forms of these drugs at high concentrations.” The authors did not discuss the implications of the metabolism of **1**.

### COVID-19^27^

More than 10,000 compounds were assayed by high-throughput screening, and **1** was identified, together with five other compounds, to be a potent inhibitor of the main protease (mPro) of SARS-CoV-2 coronavirus. The molecule of **1** was shown to be able to bind in the substrate-binding pocket of the protease. The IC_50_ of **1** was about 9 μM. The authors did not discuss the physiological relevance of the results. As another work showed, **1** is able to inhibit a panel of viral cysteine proteases but not the virus replication in cell culture; furthermore, the presence of the reducing reagent 1,4-dithiothreitol abolished the inhibitory activity of **1** toward the enzymes, thereby demonstrating the basic chemistry of the molecule.^28^

### Sepsis^29^

In this publication, **1** potently protected mice against septic shock—and the potency of the drug increased with copper supplementation. The authors interpreted the effect as “stabilization” of **2** by copper, which makes no sense from the chemical point of view (see Figure 1). **3** is not a “stabilized” form of **1**; rather, it is a totally different chemical entity. Surprisingly, the authors did not use the “stabilized” **2** in experiments with cells, but only **1** itself. **1** inhibited pyroptosis in cells with an IC_50_ above 7 μM, and after the addition of copper ions, the IC_50_ decreased dramatically to 0.41 μM. Most probably, the effect is caused by **3**.

## Conclusion

The main argument, still recurring in the scientific literature, for using **1** in biomedical experiments is that the drug has been used in the clinic for a long time and is deemed to be safe. That is the difference between **1** and similarly promiscuous chemicals used in biomedicine, such as curcumin.^30^ (Another contrast between the two compounds is that **1** has shown promising results in randomized and blinded clinical studies as being repurposed for cancer.) **1** could be immediately used off-label in real patients (e.g., compassionate use)—indeed, I have read in the mass media in my country about a doctor treating people having COVID-19 with **1**. That is a reason to be even more cautious with statements about the ability of **1** to treat a disease...and still more cautious if considering the basic chemical properties of the molecule.

In fact, **1** is a highly reactive prodrug. Any *in vitro* experiments with **1** must take into account its reactivity, its metabolism, and that only concentrations under 0.3–0.4 μM are physiologically meaningful. Because of its reactivity, **1**, especially at high concentrations, may react with hundreds of proteins in the culture media and in cells, creating the illusion of being a panacea *in vitro*. Moreover, *in vivo* experiments should also be accompanied by a clear demonstration of the active molecule in the body (e.g., **3**) and its mechanism of action *in vivo* and at physiologically reachable concentrations *in vitro*. When **1** is shown to be active in an *in vivo* model of a disease, it should be seriously discussed how it could be possible that one drug has such a “panacea-like” activity.

I suggest that editors, reviewers, and readers in general ask two fundamental questions while reading any paper discussing the repurposing of **1**: (1) Are the authors working with a concentration of the molecule that is physiologically meaningful and, if the rationale of the research is to repurpose an already approved drug, has the molecule any known pharmacological activity in human patients? (2) Do the authors really expect the same mechanism of action *in vitro* and *in vivo*—do they expect, for example, **1** to be present in the tumors? Or, if not **1**, which of its metabolites?

The ultimate goal of medicinal chemistry and biomedicine in general in the case of repurposing of **1** should be the identification and exploration of stable and potentially active metabolites of **1** in humans—and their mechanism of action at a physiologically achievable concentration. The biggest challenge, hence, is to overcome the obsession with **1** itself and the temptation to produce new articles and create new scientific output by testing **1** on more and more proteins and diseases of interest.
